# Mediating scarcity in pandemic times: an ethnographic study on the prevention and control of SARS-CoV-2 infections during the emergence of the corona crisis in the Netherlands

**DOI:** 10.1186/s12913-022-08843-0

**Published:** 2022-11-24

**Authors:** Jenske Bal, Bert de Graaff, Margreet C. Vos, Roland Bal

**Affiliations:** 1 Erasmus School of Health Policy & Management, Erasmus University Rotterdam, Rotterdam, the Netherlands; 2 Dept. of Medical Microbiology and Infectious Diseases, Erasmus MC, Rotterdam, the Netherlands

**Keywords:** Infection prevention, Covid-19, Ethnography, Resilience, Guidelines

## Abstract

**Background:**

In this paper we explore how staff involved with infection prevention managed the emerging COVID-19 crisis in the context of scarcity of Personal Protective Equipment (PPE), focussing specifically on the (re)writing of guidelines. We conceptualize guidelines as ‘mediating devices’ as they translate between evidence and clinical practice, between management and the workplace, as well as the different values embedded in these domains. It is this mediation, we argue, that adds to the resilience of healthcare organizations. The setting for this research is an elite academic hospital in the Netherlands during the emergence of the COVID-19 pandemic.

**Methods:**

We conducted non-participative observations, semi-structured interviews, and document analysis during the emerging pandemic (March–July 2020). We observed meetings from the crisis team and the unit for infection prevention (210 hours), interviewed members of these teams (21 interviews) and analysed guidelines and flowcharts concerning infection prevention, as such collecting a unique and rich qualitative dataset. Analysis was done through thematic coding.

**Results:**

Our results show the writing and rewriting of guidelines as a fundamental characteristic of dealing with scarcity and adding to resilience. We found three main practices our research participants engage in while trying to manage the uncertain situations emerging from the scarcity of personal protection equipment. The first practice we observe is defining safety; dealing with different perspectives and experiences of what ‘working safely’ means. The second entails the anticipation of scarcity by which our participants aim to control the situation through monitoring, research and creating scenarios. The third practice we observe is finding new ways to use PPE that is available, by experimenting and tinkering with the material.

**Conclusion:**

Infection prevention guidelines are crucial in managing the emerging crisis. We discuss how the writing of guidelines mediates between different settings, timeframes, and different worlds of quality. Through (re)writing there is a constant negotiation and discussion with the various actors about what works, and there is a continuous adaptive attitude. At the cost of a lot of work and struggle, this creates a resilient and inclusive work environment useful in a long-lasting crisis.

## Background

As SARS-CoV-2, or new coronavirus, spread around the world, healthcare facilities got overwhelmed by COVID-19 patients. The resilience of many healthcare systems has proven to be remarkable, with healthcare organizations and professionals quickly reacting to the initial spread of the virus, setting up new facilities, and taking care of incoming patients. This has been done amid pressing scarcities—e.g., for Intensive Care Unit (ICU) beds and related equipment, medicines, tests, healthcare personnel and Personal Protective Equipment (PPE), such as gowns, goggles, gloves, and masks. Managing quality and safety of care in this highly dynamic situation is challenging, to say the least. Conditions of care that are normally there usually were missing, and care organisations and professionals had to find ways to cope with this new situation. Moreover, little was yet known about the virus, its behaviour and effects causing many uncertainties.

Infection prevention and control have become a core and routinized practice in most Western hospitals. Many guidelines exist in relation to e.g. isolation of patients, protection of healthcare workers, and material infrastructures. Still, no country has been prepared for the magnitude of the flow of patients in the wake of the spread of coronavirus, which has led to shortages in almost all respects, often aggravated by lockdown policies of nation-states, which have caused delays in production and transport as well as international competition for scarce resources [1]. For hospitals, this meant they had to find ways to cope with the effects of scarcity in relation to quality and safety of care.

In this paper, we focus on ways in which a large academic Dutch hospital has coped with the emerging crisis in relation to quality and safety of care during the first ‘wave’ of the pandemic. We do so based on in-depth ethnographic research of the work of the crisis teams in the hospital, with a specific focus on infection prevention. With this, to the best our knowledge, unique and rich qualitative dataset we aim to contribute to the evolving body of literature within social science research to the COVID-19 pandemic and especially to the ways health providers dealt with the challenges the pandemic posed to them. To do so, we consider guidelines as mediating devices that, in their (re)writing, allow reflexive space for both experimentation and accountability. Such mediating role, we argue, adds to the resilience of healthcare organisations.

Our ethnographic research offered unique insight into a crisis organisation of an academic hospital: we were able to follow crisis management up close, as ethnographers participating in crisis team meetings and following infection prevention staff in their work. During the first weeks of the pandemic, from early March to April 2020, scarcity of PPEs was one of the main issues in the Netherlands. Questions like ‘who is to wear which types of masks?’, and ‘can we safely reuse PPEs?’ were at the top of the agenda and needed answers quickly, despite many uncertainties surrounding those questions. Academic literature written about scarcity during the COVID-19 crisis covers the ethical considerations that should be taken into account when materials are scarce; who should have priority [2, 3], how we should divide PPE worldwide, and what strategies hospitals might pursue in procuring PPE [1]. Those are important questions, but they are somewhat detached from practice and politics at the sharp end of care delivery during an acute crisis such as an emerging pandemic. Instead, we examine how scarcity is experienced, perceived and worked with on the ground, that is, within clinical practice.

We do so by reflecting on how resilience can be *done* in times of crisis. In the organizational science literature, the concept of ‘resilience’ is often used concerning coping with uncertainties to describe how good functioning crisis management works. Weick & Sutcliffe describe resilience as “the capability to detect, contain, and bounce back from those inevitable errors that are part of an indeterminate world,” arguing that resilience is “a combination of keeping errors small and of improvising workarounds that keep the system functioning” [4, 5]. But improvisation is not enough as actions also must be accounted for, especially in public service organisations such as hospitals. This then also means that action, or implementation, must be monitored and made accountable and negotiable. Resilience in this perspective is based on a combination of improvisation and monitoring, which in public administration is referred to as experimentalist governance [6]. Especially in times of crisis when uncertainties are high and scarcity ubiquitous, experimentation and monitoring are necessary to find out what works. Moreover, scarcity might come at the expense of maintaining the quality of care and thus conflict with existing regulations and accountability relations. At the same time, however, such experimentations and improvisations have to be accounted for—to healthcare workers, to patients, managers and external agencies—and accountability often comes in the form of standards that lack flexibility [7]. The challenge for hospitals becomes how to deal with these seemingly contradictory demands. Within the resilience literature, this tension between standards and working practices – sometimes referred to as work-as-imagined versus work-as-done – can be overcome by the development of reflexive practices in which contradictions can be negotiated [8]. We take the activity of the writing and rewriting of guidelines to deal with scarce PPE as one such reflexive practice – an extreme case of (potential) resilience considering the unprecedented uncertainties facing hospital staff.

This perspective allows us to explore the tension between experimentation and accountability, as we focus on the negotiation through writing guidelines for infection prevention policy. Hence, we see these guidelines as practical devices that help in dealing with conflicting demands. In the words of sociologist Bruno Latour, we argue that guidelines function as ‘mediating devices’ rather than as ‘intermediaries’ [9], as they allow for the translation and articulation of different perspectives and valuations. Guidelines in this sense are not neutral devices but mediate between evidence and clinical practice, and between management and the workplace [10]. In looking at guidelines, we focus on the practice of writing and rewriting [11]. As Callon described, with reference to Weber’s seminal work on bureaucracy, writing and rewriting guidelines is a way in which organizations learn. It is a collective practice, done through discussions and conflicts between its writers and users (ibid.: 204). The writing and rewriting of guidelines forms a reflexive practice in which experiences, changing valuations, different layers of the organisation as well as new knowledge are discussed and translated into new organisational routines. In analysing the (re)writing of guidelines, we, therefore, focus not only on the makers of guidelines, but also on their interactions with potential users, having an eye for the experiences, interests, values, and emotions involved. Focusing on guidelines thus allows us to follow the ongoing translations and articulation of clinical work as well as discussions about the different types of knowledge and values embedded therein and the uncertainties that are intrinsic to crisis management. Written guidelines are, seen in this way, a crucial part of ‘uncertainty work’ during the emerging pandemic, allowing for the temporary stabilization of knowledge and entities to allow for clinical practice to continue [12].

In the results section, we examine how uncertainties are mediated and accounted for during an emerging crisis through the writing and rewriting of infection prevention guidelines. Guidelines and flowcharts in the hospital we studied were updated daily, reflecting rapid changes in the development of the pandemic, national and regional policies, the availability of materials and personnel, clinical studies and experiences from clinical and other teams about ‘what works’. The question that guided our research was: *How do infection prevention guidelines mediate and account for uncertainties concerning the quality and safety of care during the emerging COVID-19 crisis?* Below, we first describe the ethnographic methods used for this study. Then, we show our results, using excerpts from our observations and interviews. We analyse three ways our participants were coping with uncertainties through (re)writing guidelines: ‘defining safety’, ‘anticipating scarcity’ and ‘tinkering with quality’. In the discussion section, we reflect on the uncertainties caused by scarcity, on the practices to cope with scarcity and on the mediating role of guidelines.

## Methods

### Research setting

This research is part of a broader program into the management of the pandemic [13]. The setting for what proved to become our case-study of resilience in practice is a large academic hospital in an urbanized region in the Netherlands. At that time this hospital was developing a leading role in the regional and national management of COVID-19 in the Netherlands. Dutch hospitals are private, not-for-profit organisations that function within a system of ‘regulated competition’ [14]. For acute care, however, regional consultative bodies have been established (in Dutch: *Regionaal Overleg Acute Zorgketen*, or ROAZ) that coordinate acute care within a region. University hospitals are leading in the ROAZ and thus became even more relevant in organising and managing the COVID-19 crisis. Next to hospitals, ROAZ members include GPs, ambulance services as well as actors from long-term care such as nursing homes and mental health services.

Within the hospital, a Crisis Team (CT) was established by the Board of Directors at the start of the pandemic (see also: [5]). During the first phase of the pandemic, the CT met on a daily basis. An Outbreak Management Team for Infection Prevention (OMT-IP) already started to meet in January when the threat of COVID-19 became apparent in the Netherlands and was chaired by the head (MCV) of the Unit for Infection Prevention (UNIP). The UNIP is an operational unit that consists mostly of infection prevention experts, who at the time of our research were all sitting together in one big office space. UNIP is responsible for writing the COVID-19 guidelines*.* The head of UNIP (MCV), who was the chair of OMT-IP, formed the link to the CT, as she was a member of the CT as well. In the frequent OMT-IP meetings, healthcare professionals from the hospital, such as infection control practitioners, a pulmonologist, an intensive care specialist, the head of the emergency department, managers of clinical departments, facility managers etc. were members. OMT-IP discussions and advice were discussed in the CT by the head of UNIP, including updates on the latest developments in epidemiological data, data for diagnostics, and hospital purchases. Information about the status and in-house stock of essential materials, such as personal protection equipment (PPE) were also given regularly.

### Data collection

The main methods used for this research are ethnographic, including non-participating observations of the CT, OMT-IP and the team meetings of the UNIP. The time period of observations was between March 5 and April 30 2020 when the pandemic just started in the Netherlands, with the first patient being detected at February 27. During these observations (210 hours in total, resulting in 520 pages of written notes) we closely observed the interactions between members of the board, hospital staff, crisis managers and support staff. We often engaged in informal conversations with members of the UNIP. All observations were written down, first in jot notes and then in full version, quickly after observation hours. Informal conversations were held with members of all teams to ask questions about the course of events we did not immediately understand. Moreover, at UNIP we did some ‘interviews to the double’ [15], sitting next to workers at UNIP (safely distanced), asking them to explain what they were doing in relation to infection prevention policies as they were doing it. Because of the risk of infection and the scarcity of PPEs we were not able to do observations in the clinical departments. One of the authors (MCV) was however frequently attending to these departments, as such also informing our writing. After the first wave of the pandemic had somewhat subsided in the Netherland, we started formally interviewing people from all teams we had observed. 21 interviews were performed all of which got transcribed verbatim. The interviews were conducted in Dutch; only the quotes we use are translated into English by the authors. The main purpose of the interviews was to reflect together with the respondents on the previous months and to get extra insight into certain discussions that were held during the meetings we observed. The questions focused both on the development of the crisis from an organizational perspective and personal experiences. We also gathered and analysed guidelines and flowcharts. As these changed frequently, we collected the latest version every week. We received minutes and attached documents for every meeting, and we documented news messages which were shared on the internal online forum. Furthermore, we organised three informal meetings to reflect on the preliminary results with several members of the crisis teams.

### Analysis

Data was analysed abductively, meaning we made several rounds of iteration between the data and theoretical concepts useful in understanding what we found [16]. For the analysis, we focus on the process of writing infection prevention guidelines. In observing this, we were particularly interested in the ways in which scarcity, uncertainties, and valuations in relation to PPEs were handled in the discussions and how they were decided on. Whilst we went into the observations without a clear theoretical focus, we discussed our observations on a regular, sometimes daily basis, slowly coming to more overarching themes. The themes we developed in the first instance were ‘creating and bridging distance’, ‘managing uncertainty’ and ‘doing scarcity’. These themes were later used in analysing the observation notes and interviews, through the practice of coding (using Atlas.ti) in which key terms, concepts and examples are recognized. For the analysis of the guidelines, flowcharts and news messages, we carefully analysed how certain issues got framed, and how guidelines changed over time. The minutes of the meetings served as a reference to see how certain decisions were made and formulated. Through the analysis and after more focused observations thereafter, we came to the three themes we will develop further in the result section.

### Ethics

The research plan was approved by the ethics committee of Erasmus University Rotterdam (IRB2020–08 Bal WMO, approved on 25/03/20). We received general consent from the Board of Directors for attending the meetings. For interviews, participants signed an informed consent form. All methods were carried out in accordance with relevant guidelines and regulations. The attendance of the researchers in the meetings appeared to not have that much effect on the respondents as often the participants of this study were absorbed by their work and used to others walking in and out of offices.

## Results

In this section, we discuss our findings concerning the ways our research participants dealt with the scarce availability of PPE. The head of the UNIP explains that this was the first time they “had to work in scarce times” (Interview head of UNIP, 08-06-2020). For certain materials, especially for medical masks, there was often a horizon of 48 hours, while the demand in the hospital grew. As expressed by one of the virologists: “We, as rich Dutch people are used to pushing one button, and then it’s there, now we were confronted with ‘yeah soon it is not there’. And of course, that is a huge tipping point.” (Interview virologist, 30-04-2020) To ensure the safety of employees and patients on the one hand, but to deal with scarcity on the other, plans were made to regulate the use of PPE. What ‘working safely’ means, however, was a much-contested issue. Scarcity brings the question of distribution and rationing, who gets what, when, how, and who gets to decide on these matters. In the following sections, we discuss how our respondents dealt with these questions by writing and rewriting guidelines in relation to defining safety, anticipating scarcity, and tinkering with quality.

### Defining safety

Working in an environment in which there is a new and constant prospect of scarcity created uncertainty for many healthcare workers in defining what it means to work safely. Some healthcare workers started to doubt whether the materials at hand would give them the right protection. As the head of the UNIP department explained, many healthcare workers in the hospital realised only when the corona pandemic started in earnest in the Netherlands that they were at risk to be infected as well. Before, with previous infectious diseases, they executed protective measures mainly on behalf of the patient’s safety, “and then it is something outside of yourself” (Interview head of UNIP, 08-06-2020). COVID-19, however, was experienced as a risk not only for patients but for healthcare workers too. It became the responsibility of the department for infection prevention to decide what working safely in the hospital means and to communicate these decisions to all employees of the hospital – from cleaning staff to the board of directors. Given the many uncertainties related to infection routes taken by the novel coronavirus and the levels of protection needed in specific situations, they needed to keep explaining that people would have to “learn to live with this kind of uncertainty. […] We can never guarantee a 100% risk reduction.” (Interview head of UNIP, 08-06-2020).

In the OMT-IP meetings, the interdisciplinary team constantly weighed the risks of what according to them working safely means. In one meeting for example a discussion arose about whether people walking in the hallways of the, just now created COVID-19 departments, would have to wear masks, as aerosols or even droplets might be present in the adjacent hallways:

“We should explain that we think about what is safest. Putting on a mask in the hallway is not a good idea, there might even be a great risk of infection as people take it off, touch it, and put it on again. Every time we must consider what has the least risk. Right now, that includes that people do not wear a mask in the hallway.” (Observation OMT-IP, April 6, 2020).

In another OMT-IP meeting, a request from the cleaning staff in the hospital was discussed. The cleaning staff argued they did not feel safe enough to enter a room in which a COVID-19 patient had been, without wearing an FFP2 mask (used for protecting against aerosols). In an OMT-IP meeting in which this issue got discussed, it was stated that for cleaning staff a mask would not be necessary at all. Someone proposed to give the cleaners another mask, offering less protection than the FFP2 version; the ‘surgical FFP1 mask’, just to make them feel safe. Another participant in the meeting mentioned that by doing this you would trick them into thinking that they are safe, just to let them continue their job. Someone else answered: “yes, but without it, they would not go into the room at all.” (observation notes, March 19, 2020) Not giving in to the wish of the cleaners, who have an important role in infection prevention as they keep the working environment hygienic, would create the risk of them refusing to continue their work. However, submitting to the wish of the cleaning staff would create the risk of running out of masks. Just like with clinical staff, there is an inherent power dimension at play here; but whilst clinical staff could voice their concerns, cleaners had no spokespersons to represent them directly in the OMT-IP. Their power was not unsubstantial however as refusing to clean rooms would create serious difficulties for the hospital.

A few weeks into the pandemic, members of the UNIP started to make short instruction videos targeted at specific groups of staff in which they explained the guidelines and argued why working under these circumstances is still safe. Through such educational materials, the UNIP staff hoped to reduce hospital staff’s anxieties, incomprehension, and uncertainty. Furthermore, each day representatives from the UNIP walked around the COVID-19 departments to think along with professionals on the arrangements to work safely and to gather feedback, which they then used in rewriting guidelines. During these rounds, they observed behaviours in the clinics to see how the social and material infrastructures they proposed were working in practice. Guidelines concerning PPEs were supposed to be carried out exactly as they were stated – to minimise the risk of infection while not using too many or inappropriate masks – so walking around the departments also served to monitor whether this happened according to the guideline. Walking around functioned also as a way to account for the guidelines: members of the UNIP patiently and repeatedly explained why they choose certain measures over others. On the other hand, such rounds helped the UNIP team to see if guidelines actually worked in practice and helped them in revising guidelines to make them a better fit with practice.

Some members of UNIP were stationed at the UNIP office to pick up the phone and answer questions by medical staff in the hospital. These questions also provided UNIP with feedback on the guidelines and sometimes it became clear which issues needed more explanation or change. Questions from medical professionals could be rather critical. The head of the UNIP explained that medical professionals would also consult the guidelines made by their national-level medical specialist association: “we had a knowledge gap, and this gap got picked up by each association separately” (Interview head of UNIP, 08-06-2020). Consequently, many specialists within the hospital wanted to follow the guidelines made by their medical specialist associations, instead of the ones drawn up by the UNIP in their hospital. According to one member of the UNIP, this “caused a lot of trouble”, as there had to be a constant debate about why certain guidelines were preferred over others. “I think it cost 50% of the [time of the] entire crisis management.” (Interview clinical microbiologist, 29-05-2020).

Through the collective practice of writing guidelines, the different risks, uncertain feelings, experiences and power imbalances regarding scarcity got mediated. We found UNIP staff constantly balancing between minimizing the risk of infection for healthcare workers and patients amidst times of (perceived) scarcity and submitting to the wish of many healthcare workers and hospital staff to always use the highest quality protective measures. Mediation happens here between the different perspectives and experiences of safety by people on the work floor, professionals within the hospital, national professional associations, and hospital policy as well as between different valuations of work (e.g., the cleaning staff). The collective writing of guidelines we see in the OMT-IP meetings allows for an open dialogue, which then gets communicated and accounted for by taking rounds around the COVID-19 departments, allowing for an adaptive governance approach.

### Anticipating scarcity

At the beginning of the pandemic, knowledge lacked about the nature of the coronavirus, the effects of the virus, and what the best protective measures were to minimise infections, resulting in different ways of sense-making by medical staff and crisis managers [17]. Guidelines got based on previous experiences with other infectious diseases, and on the expertise of available professionals. As much knowledge lacked in the beginning, the guidelines could not always be based on existing empirical evidence on this specific virus, and often got based on the tacit knowledge of the infection prevention experts and their network (cf. [18]). Understanding the new virus research was a collective effort that caused many discussions within the UNIP and with the interdisciplinary team meetings of the OMT-IP. When these experts noticed consensus could not be reached, occasionally additional research was performed. For example, a literature study was done on ‘aerosol-generating procedures’ after intense discussions. In the beginning “we told everyone to use an FFP2 mask during aerosol-generating procedures, but nobody really knew what these were” (Interview expert infection prevention, 15–05-20). People started to ask whether operations they were performing could also be seen as ‘aerosol-generating’, but “no one knew it exactly and nobody dared to make decisions about it,” because it would have enormous consequences for how many masks and goggles had to be used (Interview expert infection prevention, 15–05-20) and on the other hand, safety for healthcare workers.

The knowledge produced by professional and national governmental associations, such as the Dutch Public Health Institute was closely monitored and consulted, and where possible contributed to (the guideline on aerosol-forming activities for example became part of the guidelines of the Dutch Federation of Medical Specialists). These national guidelines then got translated to the context and situation of the hospital. An example of this becomes apparent in a meeting of the crisis management team of the hospital. The head of the UNIP shared with the participants that the Public Health Institute had published a report that a surgical mask would suffice for operations in which usually, before COVID-19, a safer FFP1 mask would have been used. The head of UNIP argues to execute this measure in the hospital as soon as possible; this might help prevent a real shortage of masks. She anticipated here the worst-case scenario in which there are no masks left at all – and care-provision would potentially come to a halt, “but we should communicate [this decision] carefully, as people do not want that. We do not want to have to implement this measure in times of scarcity, so we want to ask if you all agree to start implementing it slowly starting now.” The members of the CPT decided that, first, clear predictions of the stock of masks in the hospital had to be made, and if necessary, indeed gradually introduce the use of the surgical masks (Observation CPT, March 31, 2020). In an interview with a UNIP member, looking back on this very moment, the respondent points to it as one of the tensest moments in the whole crisis, as this would mean staff would have to work under precarious, unsafe, conditions: “are we not going to make people terribly anxious about it?” (Interview expert infection prevention, 15-5-2020)

**Fig. 1:**
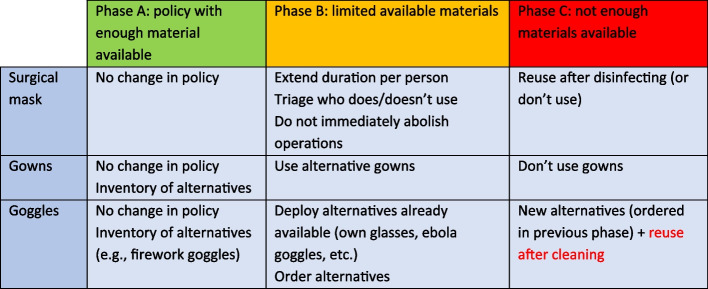
Scenarios for materials. Translated to English: original and extended version was shown during CPT in Dutch (March 13, 2020)

To know how many PPE were available, making inventories and monitoring stock levels started to become a routinized practice. Every CT and OMT-IP meeting, an update was provided by a representative of the purchasing department about how many materials were presently in stock. At first, these numbers were provided for the coming 48 hours – a testament to the tense situation. Later this changed to 7 days. This way, when the moment would come when there would not be enough materials left, this would be noticed immediately and could be acted upon. Scenarios got formulated planning out how to use PPE based on different levels of scarcity – with the worst-case scenario still being very much experienced as a real possibility by our participants. The UNIP devised a scenario for three phases – green, orange, and red – each with a different quantity of stock. Figure 1 describes for a few protective materials what should happen in which phase. As the head of the infection prevention department explained, the column marked red indicates a different way of working: “when materials are scarce, we need to work differently, less safe for example.” (observation CT, March 13, 2020)

Scarcity in this sense is translated into a temporal issue, as for each item, there is a limited supply for a demarcated period. Scarcity gets projected into the future. Through creating these scenarios, the UNIP tried to be prepared for any eventuality. By laying down these scenarios, which then got translated into guidelines, the UNIP took control over possible situations of increased scarcity in the future. Moreover, it allowed for healthcare staff to be prepared for any eventualities. Mediation is visible here between different settings and different time frames. By making these scenarios, UNIP-staff could account for the fact that a plan was already formulated in case the situation occurred. Once an alternative plan had to be deployed, the UNIP updated this in the guidelines and flowcharts to be uploaded on the internal online infrastructure as well as in the educational films made for staff.

### Tinkering with quality

The scarce availability of PPE sometimes meant that products got purchased that did not meet the usual quality standards. The UNIP staff then had to find ways in which these materials could be used and still meet their quality standards and guidelines. When a new type of FFP2 mask would arrive, a ‘fit-test’ was performed to see how many virus particles would go through the materials and what would be the best way to wear the mask. We analyse this experimental way of mediating between materials and quality standards to be a *tinkering* way of finding solutions [19].

During an OMT-IP meeting, the UNIP staff announced the arrival of a new mask fabricated in China, which did not meet the EU standards but had to be used anyway as no other masks were currently available. A member of the UNIP explains it was very difficult to make the masks fit: “apparently we have a different head than the Chinese” (in the end a model of a ‘general’ Dutch head shape was even sent by the national coordination centre to manufacturers in China to improve the fit—see [20] on the implicit racism in craniofacial research). The UNIP member goes on to explain a special clip with elastic bands which could be attached to the back of the head was ordered, improving the fit of the mask. However, “it turned out it is difficult to then take the masks off because the bands get caught up in hair and earrings.” The guideline had to be rewritten, stating people with long hair should wear a cap, to then lash up the strings with a clip over their cap (Observation OMT-IP, April 6, 2020). UNIP staff found this out by trying the mask on themselves and letting it try out by healthcare professionals at a few departments.

**Fig. 2:**
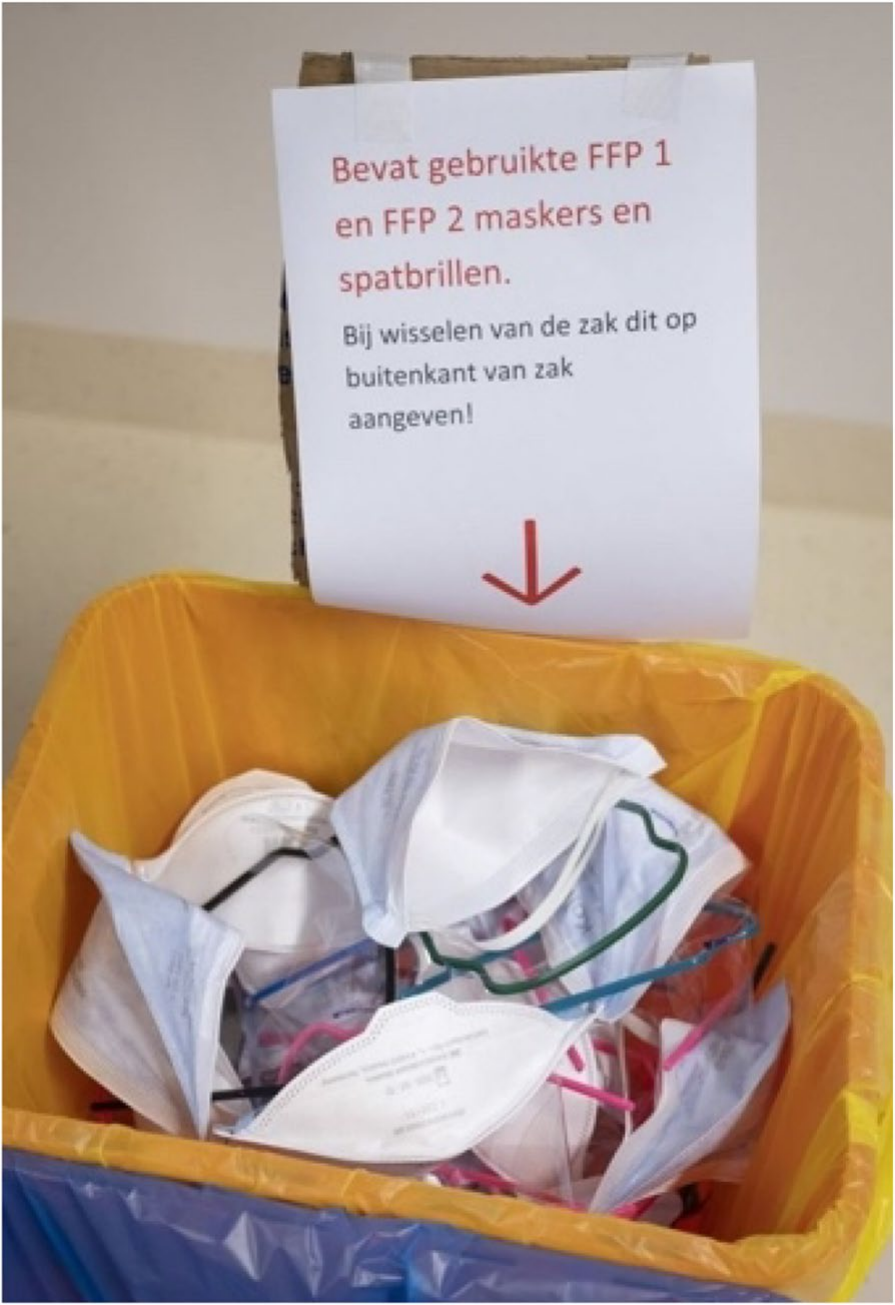
Basket for used facemasks

When certain materials were about to get scarce, the UNIP staff also thought about alternative options such as reuse. For goggles and masks, a team of experts in sterilisation materials started to explore the options to sterilise used materials and baskets to collect masks were distributed to Covid wards (Fig. 2). During an OMT-IP meeting, people working on this gave a presentation about their research. They told the whole group about the technicalities of doing sterilization tests, one of them being a ‘steam test’. Some of the surgical masks came out all shrivelled making them useless for reuse. For other masks, steaming worked very effectively; the masks worked almost equally well in stopping virus particles. One of the main challenges for sterilisation is the huge diversity of masks that got supplied. Participants of the meeting questioned when these sterilized masks ought to be used. The answer UNIP staff provided them was to wait and see when it became necessary to start using them (see Fig. 1). Defining the moment when not enough material was available – and thus reuse was needed – proved to be dependent on the perspective one took, as became apparent during a discussion in the OMT-IP.

The representative of the purchasing department states: “we still have enough masks here”. The head of the UNIP department acknowledges this but argues that, although this might be true, it is important to continue exploring all the possibilities to reuse PPE – not only for potential future situations within the hospital, but also in relation to other care organisations in the region: “later we will get blamed: ‘you use a lot of masks, then also reuse them.” The person responsible for the purchase again states that “we will have enough masks here,” to which the head of the UNIP responds: “We might have enough boxes with masks here, but we have to see it in the context of the region as well” – pointing to the broader role this hospital plays as the largest hospital in the region. (Observation OMT-IP, April 10, 2020).

Sterilizing masks here becomes a more political action, anticipating accountability to healthcare organisations in the region, rather than just a matter of safety within the hospital; with the university medical centre leading in the region, it also had to show responsible use of face masks as all healthcare organisations were struggling with scarcity issues. And this greater responsibility then fed back to deciding on the moment at which scarcity would set in.

Acting on (anticipations of) scarcity, any changes in standards of quality became mediated through experimenting and tinkering with the materials by UNIP staff. Through trying out different ways to adapt the materials to fit the healthcare practices, the UNIP team focused on the quality and right use of materials. This process of tinkering and experimenting with materials did not only happen for the standards of quality within the hospital. Although there was always the prospect of scarcity in the near future (a few days), there has never been an actual shortage in PPE on the level of the wards in this hospital. In other healthcare facilities, however, particularly in Dutch nursing homes and home care, the situation proved more dire. This meant decisions made in the hospital needed to be accounted for in meetings with other healthcare organisations in the region, which can be seen as an anticipated form of accountability. Mediating through guidelines thus not only was targeted at relations between managerial and clinical departments but also between organisations in the region, through which the hospital showed its responsibility to broader concerns.

## Discussion

We zoomed in [21] on the (re)writing of guidelines – happening in and around many meetings and educational activities by UNIP staff and discussions in OMT-IP and CT meetings. We found three main themes in those (re)writing practices: defining safety, anticipating scarcity, and tinkering with quality. All these themes included both managing uncertainties in terms of the evolving knowledge about the virus and protective measures and valuations of who needed protection. We found how (re)writing served as a very deliberative and reflexive practice, in the sense that uncertainties and valuations were constantly discussed and monitored. This then enabled a resilient approach to infection prevention, anticipating possible risks associated with the scarcity of protective equipment, uncertainties in infection routes as well as uncertain reactions of healthcare workers and other organisations in the region. The writing and rewriting of guidelines proved central in this (cf. [22]).

This approach of (re)writing was very much bound to the specific context of our case-study, this particular academic hospital; arguments of staff had to be accounted for in the rewritten guidelines but also had broader references as regional responsibilities were translated into tinkering practices. Moreover, the process of (re)writing also resonated throughout the Dutch healthcare system as guidelines created in this hospital sometimes moved to other hospitals, and other settings (healthcare sectors) – to be translated yet again to different contexts and practices. For instance, the head of UNIP (MCV) was also heavily involved in national level discussions. As soon as national guidelines were developed, these became the base of adapting the early made guidelines of the hospital. Through the writing and rewriting of guidelines that bring together “the different logics that coexist in professional practices” ( [23]: 1086), mediation becomes visible.

Mediation, we have shown, is not a neutral activity. In bringing together the different ‘logics’ of infection prevention, hospital policies and clinical practices, choices have to be made that favour specific actors and arguments above others. For example, to provide doctors and nurses with masks to be protected when in contact with patients, masks might no longer be available for other staff in the hospital, like the cleaning staff – which then had to be accounted for. The cleaning staff’s resistance necessitated further mediation, discussing what level of protection might be necessary to accommodate their fears. Valuing different kinds of work thus is an integral part of the (re)writing of guidelines. Possibilities for resistance, this example shows, give rise to reflexive practices. This is also expressed in the rounds the UNIP staff made daily around the clinical departments through which information about the ways in which the guidelines were implemented was collected. These observations were fed back into the guidelines, leading to changes in guidelines or communication strategies. Accountability, as stated in the introduction, usually comes in the form of standards that lack flexibility [7]. In a crisis, however, accountability can be seen as an ongoing process. Because of the reflexive loop in this constantly changing situation, new knowledge becoming available and observations and feedback about ‘what works’, guidelines constantly get rewritten. Such is reminiscent to how Mol introduced ‘tinkering’ as attentive experimentation in the context of care [19]. Good care requires continuous tinkering with conflicting valuations to arrive at a seemingly stable and suitable material, emotional and relational management of – in this case – safety of patients and staff in the context of scarcity (cf. [24]).

We find mechanisms of mediation on different dimensions. Mediation happened between different settings on a local, regional, and national level. This became explicit especially when knowledge became available on these different levels, or when ways to deal with scarcity got negotiated between different healthcare organisations. For instance, a regional working group on sharing guidelines, experiences on implementation and shortage was installed by the head of the UNIP (MCV). Framing the geographical scale at which scarcity plays a role played an important part in the mediating attempts on a local level, i.e., within the hospital.

Moreover, mediating is a temporal practice as was shown by the use of scenarios to get a grip on possible future situations. Projecting which possible futures might be relevant allowed for actions that needed to be taken in the present, for example by starting to use other (less protective) masks in the operating theatre now in preparation for a potential lack of masks in the future. Our focus on mediation showed that scarcity is not just a measurable phenomenon but rather a negotiated reality and result of a process of sense-making as well as power struggles as became apparent for example in the discussion of scarcity in relation to the regional responsibility of the hospital. Mediating scarcity in this way refers both to these sense-making practices and the work needed to deal with resulting ways of defining scarcity.

In the literature on resilience in relation to crisis management, the focus is mostly on the actions taken to monitor, respond to and recover from a crisis. Not much attention is paid to how those actions are accounted for [8]. We have shown that accounting practices – as expressed in talks to clinical staff, in discussions in the crisis teams, to other hospitals in the region as well as to regulators – are not just an integral part of managing a crisis but also crucial as they create the reflexive space necessary to make sense of and frame situations, negotiating the different values at stake and finding ways to deal with such value conflicts. This relates to questions about who gets protected (to what level) as well as to the quality and safety of care offered. Guidelines, in the literature often depicted as inflexible and based on ‘work-as-imagined’ [24], to the contrary in our case-study proved to be essential in bringing together the different worlds of healthcare professionals, faciliatory staff, crisis managers and people working on infection prevention. Moreover, guidelines proved to be not static, but very dynamic, as their almost continuous rewriting allowed for the inclusion and representation of new insights and valuations, responding to the developments of the pandemic ([18] cf.). Future research on resilience should take this dynamic role of guidelines and their relation to accountability into account.

We have been able to observe crisis decision-making from up close, which allowed us to collect unique data on the management of the COVID-19 pandemic in a hospital setting. We have found no other studies that were able to report on such detailed data. As this was a single-center study, our results are nevertheless very situated. Moreover, the centre we studied was very much on the forefront of national discussions, so observations on more peripheral hospitals might lead to somewhat different results. Although we did not manage to observe clinical work as well, where tinkering with protocols in care practices can be observed [25], and have not been able to interview all relevant actors (e.g. the cleaners), by using experiences from UNIP members (such as MCV) that performed walkarounds in the clinic, we were nevertheless able to discuss the mediating role of guidelines vis-à-vis clinical practice to some extent.

## Conclusion

In this paper, we examined how infection prevention guidelines for PPE mediate and account for uncertainties concerning quality and safety of care during the emerging corona crisis. We analysed how the infection prevention staff of an elite university hospital in the Netherlands managed the scarcities prominently emerging at the start of the COVID-19 pandemic. We showed three ways of mediating and accounting in relation to scarcity: defining safety, anticipating scarcity, and tinkering with quality. These practices together enabled a resilient response to infection prevention. The writing and rewriting of protocols showed to be constitutive of these practices.

## Data Availability

The datasets generated and/or analysed during the current study are not publicly available due to their trustworthiness but are available in pseudonymized form from the corresponding author on reasonable request. All data are in Dutch.

## Abbreviations

CT: Crisis Team
ICU: Intensive Care Unit
FFP: Filtering facepiece
GP: General Practitioner
OMT-IP: Outbreak Management Team Infection Prevention
PPE: Personal Protective Equipment
ROAZ: Regionaal Overleg Acute Zorgketen (Regional coordination body for acute care)
UNIP: Unit for Infection Prevention
